# Identifying Genetic Differences Between Dongxiang Blue-Shelled and White Leghorn Chickens Using Sequencing Data

**DOI:** 10.1534/g3.117.300382

**Published:** 2017-11-29

**Authors:** Qing-bo Zhao, Rong-rong Liao, Hao Sun, Zhe Zhang, Qi-shan Wang, Chang-suo Yang, Xiang-zhe Zhang, Yu-chun Pan

**Affiliations:** * Department of Animal Science, School of Agriculture and Biology, Shanghai Jiao Tong University, 200240, P.R. China; † Institute of Animal Husbandry and Veterinary Research, Shanghai Academy of Agricultural Sciences, Shanghai 201106, China; ‡ Shanghai Key Laboratory of Veterinary Biotechnology, Shanghai 200240, P.R. China

**Keywords:** chicken, XP-EHH, EigenGWAS, EMMAX, selected regions, functional annotation

## Abstract

The Dongxiang Blue-shelled chicken is one of the most valuable Chinese indigenous poultry breeds. However, compared to the Italian native White Leghorn, although this Chinese breed possesses numerous favorable characteristics, it also exhibits lower growth performance and fertility. Here, we utilized genotyping sequencing data obtained via genome reduction on a sequencing platform to detect 100,114 single nucleotide polymorphisms and perform further biological analysis and functional annotation. We employed cross-population extended haplotype homozygosity, eigenvector decomposition combined with genome-wide association studies (EigenGWAS), and efficient mixed-model association expedited methods to detect areas of the genome that are potential selected regions (PSR) in both chicken breeds, and performed gene ontology (GO) enrichment and quantitative trait loci (QTL) analyses annotating using the Kyoto Encyclopedia of Genes and Genomes. The results of this study revealed a total of 2424 outlier loci (*p*-value <0.01), of which 2144 occur in the White Leghorn breed and 280 occur in the Dongxiang Blue-shelled chicken. These correspond to 327 and 94 PSRs containing 297 and 54 genes, respectively. The most significantly selected genes in Blue-shelled chicken are *TMEM141* and *CLIC3*, while the *SLCO1B3* gene, related to eggshell color, was identified via EigenGWAS. We show that the White Leghorn genes *JARID2*, *RBMS3*, *GPC3*, *TRIB2*, *ROBO1*, *SAMSN1*, *OSBP2*, and *IGFALS* are involved in immunity, reproduction, and growth, and thus might represent footprints of the selection process. In contrast, we identified six significantly enriched pathways in the Dongxiang Blue-shelled chicken that are related to amino acid and lipid metabolism as well as signal transduction. Our results also reveal the presence of a GO term associated with cell metabolism that occurs mainly in the White Leghorn breed, while the most significant QTL regions mapped to the Chicken QTL Database (GG_4.0) for the Dongxiang Blue-shelled breed are predominantly related to lesions, bone mineral content, and other related traits compared to tibia length and body weight (*i.e.*, at 14, 28, 42, and 70 d) in the White Leghorn. The results of this study highlight differences in growth, immunity, and egg quality traits between the two breeds, and provide a foundation for the exploration of their genetic mechanisms.

The Chinese indigenous Dongxiang Blue-shelled chicken breed has been subject to constant attention from the poultry industry over recent decades because of its numerous excellent characteristics (Wang *et al.* 2009). In tandem with the White Leghorn—a breed originally native to Italy that has particularly desirable production attributes—the Dongxiang Blue-shelled chicken has been subject to intensive selection over recent years. Thus, due to both natural selection and persistent human domestication, both these chicken breeds have experienced huge genomic changes in their growth, immunity, egg quality, and eggshell color. Large-scale regions of the genome that exhibit marked variation caused by selective pressure are called “selected regions”; identifying the genes and/or genetic variation within these regions is key to the analysis of selection and evolution. Although Liao *et al.* (2015) revealed the presence of novel growth and egg trait variants in Dongxiang Blue-shelled and White Leghorn chickens, research on selected regions of the genome between these two breeds has not yet been reported. Identifying genomic loci under selection that correlate with particular features would be beneficial for future breeding programs, as well as to identify genes in chicken that are related to biological processes and traits of interest.

As a result of rapid technological developments in high-throughput gene analysis, it is now possible to obtain genome-wide genetic markers at low cost, enabling the evaluation of genetic mechanisms in poultry at this level. These approaches are also useful for the detection of selected regions related to specific traits via the identification of distorted patterns in genetic variation; Li *et al.* (2012) utilized chicken microarray data to detect ∼385 selected regions in White Leghorn chickens and revealed a series of genes related to egg production, metabolic, and immune response traits including *EYA2*, *NCKX1*, and *LHK2*. Similarly, Gholami *et al.* (2014) utilized 600K single nucleotide polymorphism (SNP) chips from three laying and 14 nonlaying strains to show that the genes *NCOA1*, *SREBF2*, and *RALGAPA1* are related to breeding, laying, nesting, and other traits. One strategy applied to date for studying genetic variation has been aimed mainly at the detection of selection signatures based on identified patterns of linkage disequilibrium (LD) (Ennis 2007). A series of detection statistics for these selection signatures has been developed, including extended haplotype homozygosity (Sabeti *et al.* 2002) for use in cases of recent selection, as well as Tajimas’ D statistic (Tajima 1989), which can be applied in cases of relatively ancient selection. However, methods such as Tajima’s D (Tajima 1989), the Hudson-Kreitman-Aguade test (Hudson *et al.* 1987), and Fay and Wu’s *H* test (Fay and Wu 2000) were not designed for locating genome-wide SNPs, while other methods, such as Relative Extended Haplotype Homozygosity (REHH) (Sabeti *et al.* 2002) and Integrated Haplotype Score (iHS) (Voight *et al.* 2006), are not suitable for detecting selection signatures at the genome level between two breeds. Thus, given the aims of this study, and the various statistics available to identify the signatures of positive selection from SNP data, we chose to utilize the cross-population extended haplotype homozygosity (XP-EHH) test. We also applied eigenvector decomposition combined with genome-wide association studies (EigenGWAS) and efficient mixed-model association expedited (EMMAX) (Kang *et al.* 2010) methods in this study to achieve maximum statistical power for localizing selection sources, and confirming the results of the XP-EHH method.

We also performed principal component (PCA) and LD analyses on SNPs in this study. We investigated selection patterns in the two chicken breeds, and identified several genomic regions that control traits related to growth, egg quality, and immunity. Thus, by analyzing nucleotide diversity, we aim to identify genomic regions that exhibit selection signatures and candidate genes that occur in proximity to these regions. We hope to identify the most significant indicators of selection, and thus gain further insights into the genome-wide footprints of chicken selection. We also investigated the functions associated with genes under likely selection via gene ontology (GO) enrichment analysis applying Kyoto Encyclopedia of Genes and Genomes (KEGG) annotations and pathways.

## Materials and Methods

### Preparation of population and sequencing data

Individual laying chickens were housed in individual cages at the Shanghai Xin Yang Poultry Breeding Center, Shanghai, China. We sequenced 252 Dongxiang Blue-shelled and White Leghorn chickens using the genotyping by genome reducing and sequencing (GGRS) method (http://klab.sjtu.edu.cn/GGRS/) (Chen *et al.* 2013). This method utilizes next-generation sequencing technology, and enables the effective and highly reproducible genotyping of species. We generated ∼400 million raw reads using this approach, an average of ∼1.4 million good reads per sample. We have previously reported detailed information regarding the individual chickens within our genotyping sample as well as our methods for acquiring raw Illumina DNA sequence data (Liao *et al.* 2015). In this study, we mapped raw sequence reads to the chicken reference genome (Gallus_gallus-5.0) using the Burrows-Wheeler-Aligner (Li and Durbin 2010), while SNP calling was completed using the software SAMtools (version1.6) (Li *et al.* 2009). We applied the following filter requirements for SNPs: (1) SNP test scores ≥20 (*i.e.*, an accuracy of >99%); (2) SNP calling rates ≥90%; (3) Minor allele frequency ≥5%; and (4) SNPs detected are the only ones that appear on a fixed chromosome. We then phased SNPs prior to genotyping using the software FASTPHASE (Scheet and Stephens 2006) and imputed missing genotypes using the software imputed best linear unbiased prediction (iBLUP) (Yang *et al.* 2014) for further positive selection analysis. The iBLUP software implements a method for imputing missing genotypes using identity-by-descent and LD information. This method can impute missing genotypes with greater accuracy than other commonly applied approaches, including the software BEAGLE (Yang *et al.* 2014). The SNP and phenotype data are available through the link http://klab.sjtu.edu.cn/SNPchicken/data.zip or https://jbox.sjtu.edu.cn/l/FoibdS.

### PCA

We performed PCA to collate the information contained in all SNPs applying the eigen function in the software R. Thus, we obtained eigenvectors in descending order and utilized the first two to distinguish population structure.

### LD analysis

We calculated the genotype correlation coefficient (*r^2^*) between all pairs of SNPs using the software PLINK (Purcell *et al.* 2007) (version 1.07) in order to estimate genome-wide LD, and summarized *r^2^* values at different distances by calculating means across all chromosomes.

### The XP-EHH test

The XP-EHH test was proposed by Sabeti *et al.* (2007), and utilizes the extended haplotype method and iHS construction strategy. This statistical method improves the reliability of selected region detection by introducing a group comparison strategy, expressed as follows:

$$\text{XP}-\text{EHH}=\ln\left(\frac{I_{A}}{I_{B}}\right).$$

In this expression, *I_A_* denotes the integral of the EHH statistic of genetic distance in the observed population, while *I_B_* is the integral for the reference population (Sabeti *et al.* 2007). Because this approach is comparable between the selected regions of the two groups, we applied the XP-EHH test (Sabeti *et al.* 2007) to detect selection signatures between the two chicken breeds. We used White Leghorn chickens as the observation population in this study, while the Dongxiang Blue-shelled breed was designated as the reference population. Thus, a positive XP-EHH test value demonstrates selection in the observation population, while a negative value is indicative of selection in the reference population. We calculated test values using the software XP-EHH (http://hgdp.uchicago.edu); because the empirical distribution of XP-EHH statistics conforms to a normal distribution, these can be used to calculate *p*-values based on a standard normal distribution. As positive and negative values of this statistic can be distinguished based on the two different groups, we calculated *p*-values using a two-sided test, and designated values <0.01 as outlier loci. Thus, if the distance between two outlier loci was <10 kb, we supposed that they were merged into one outlier section, while genomic segments extended on both sides of this section were defined as potentially selected regions (PSRs).

### EigenGWAS and EMMAX analysis

Eigenvectors have routinely been used in population genetics to quantify genetic differentiation across populations and to infer demographic history (Patterson *et al.* 2006; Nelson *et al.* 2008). Thus, in order to complement and verify the results obtained via the XP-EHH test, we utilized EigenGWAS analysis to further determine ancestry informative markers and loci under selection. The EigenGWAS phenotype is an eigenvector generated from GWAS data, commonly used in population genetics to characterize the structure of genetic data (Chen *et al.* 2016). SNP effects can be estimated using the single-marker regression, which is computationally much easier in practice, and is implemented in many software packages (Chen *et al.* 2016). Here, we used the following code: “ java –jar gear.jar eigengwas–bfile plink–ev 5–out plink” to get the *p* value of SNPs. “–bfile ” specifies the genotype files in plink binary format, and “–ev” specifies the eigenvectors that are used from EigenGWAS analysis. More details can be found in Chen *et al.* (2016).

Although GWASs have been used to identify numerous loci associated with complex traits, we applied EMMAX analysis in this study as it is known to outperform both PCA and genomic control in correcting for sample structure (Kang *et al.* 2010). EMMAX code for calculating the kinship matrix and more details can be found in Kang *et al.* (2010).

### Functional gene set enrichment analysis

We performed an additional analysis to further elucidate the biological function of selected regions, initially mapping candidate regions and genes using gene annotation data for chicken extracted from the Ensembl Genes 87 database (http://asia.ensembl.org/info/data/index.html). We annotated GO analyses using the KEGG as well as for candidate-selected regions using the Database for Annotation, Visualization and Integrated Discovery (DAVID v6.7). We defined a significant threshold *p*-value to be 0.05, and established a link to reveal relationships between the selected regions and characters. This enabled us to map selected regions onto quantitative trait loci (QTL) sections using data from the chicken QTL database (http://www.animalgenome.org/cgi-bin/QTLdb/GG/index). Because not all of the QTL are suitable for analysis, as the lengths of some are too large for efficient postprocessing, we removed QTL with lengths >1 Mb, and, when two or more QTL overlapped by >50%, we merged them into one larger QTL. Then we mapped the selected regions to the QTL regions from the chicken QTL database. If the QTL falls within the selected regions, or the region falls within the QTL, we define it as an overlap. The PERL script was used to conduct the above QTL-based annotations.

### Data availability

All the SNP and phenotype data we used have been uploaded to our website (http://klab.sjtu.edu.cn/SNPchicken/data.zip or https://jbox.sjtu.edu.cn/l/FoibdS). Supplemental Material, File S1 contains the results of XP-EHH analysis. File S2 contains the results of EigenGWAS analysis, and File S3 contains the results of EMMAX analysis. File S4 contains a genotype file with a total of 100,014 SNPs and a phenotype file with five columns of eigenvectors.

## Results

### PCA

**Figure 1:**
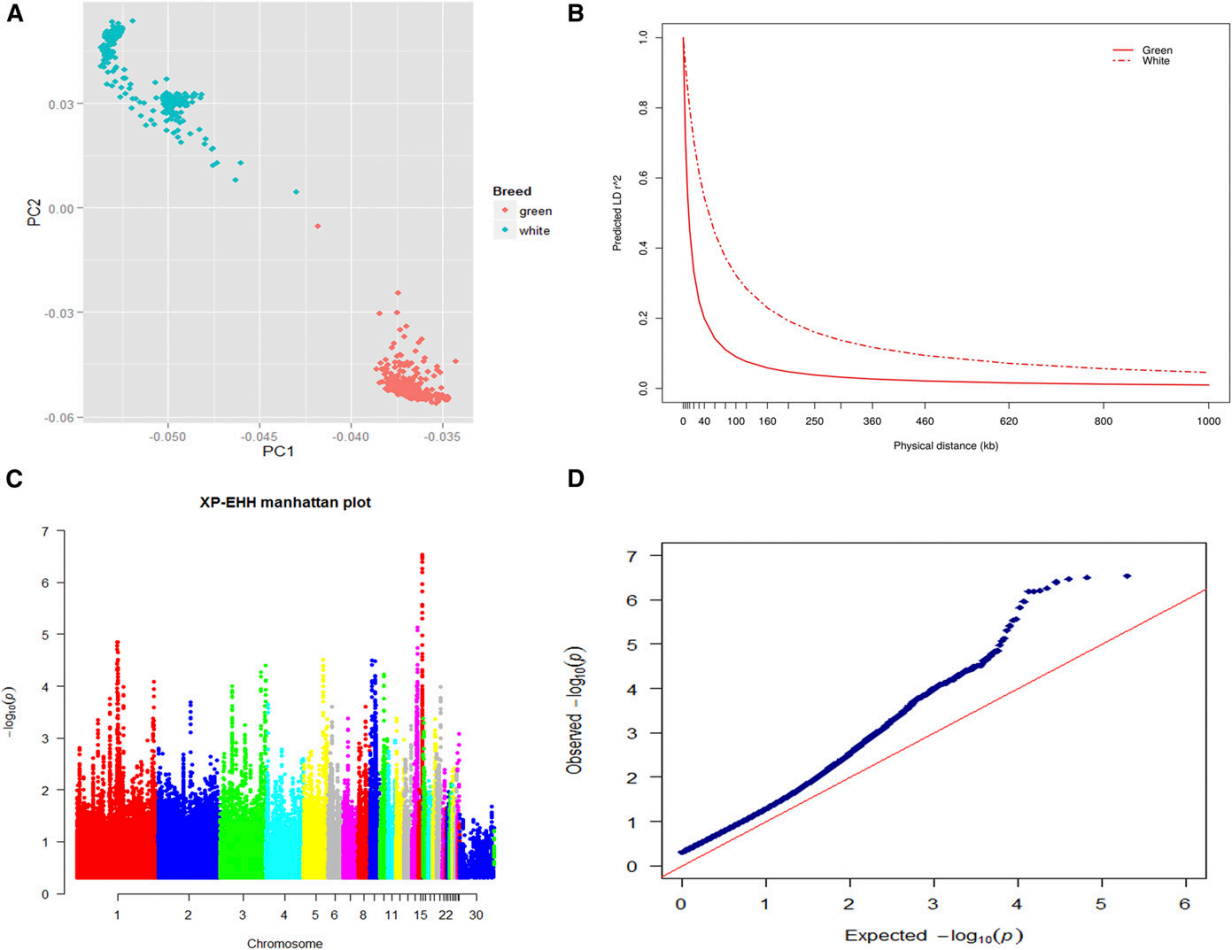
(A) PCA results for White Leghorn and Dongxiang Blue-shelled chickens. In this graph, eigenvector 1 is on the *x*-axis, while eigenvector 2 is on the *y*-axis. (B) Decay in average pairwise LD (*r^2^*) over distance between SNPs in White Leghorn and Dongxiang Blue-shelled chickens. (C) Genome-wide distribution of selection signatures detected by using the XP-EHH test. (D) Q-Q plot of *p*-values with XP-EHH test.

We performed PCA on loci from entire genotypes (*i.e.*, SNPs; *n* = 100,114) to characterize the pattern of individual samples. As shown in Figure 1A, both the first and second eigenvector (63.3% of total variance) distinguish White Leghorn from Dongxiang Blue-shelled chickens. At the same time, two strains of White Leghorn chickens can also be differentiated via the second eigenvector (14.5% of total variance).

### LD analysis

We calculated LD between all pairs of SNPs within each population; results show that LD decays with increasing distance in both breeds (Figure 1B). Average *r^2^* was higher in White Leghorn compared to Dongxiang Blue-shelled chickens between 0 and 1 Mb, while breed differences decreased as distance between markers increased. When the *r^2^* threshold was set at 0.3, LD extended to 120 kb in White Leghorn chickens, compared to 20 kb in the Dongxiang Blue-shelled breed.

### Detection and distribution of PSRs

**Figure 2:**
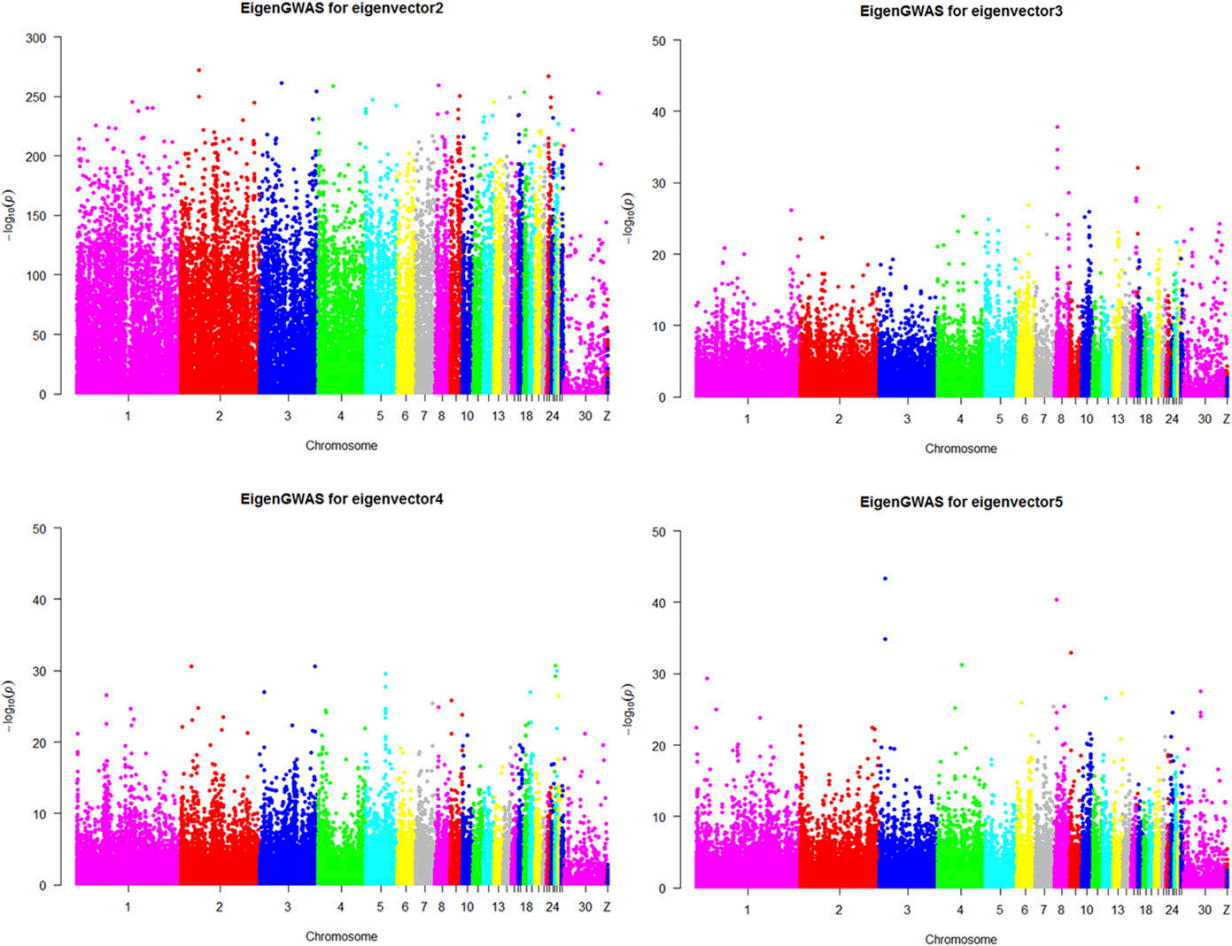
Manhattan plots generated using EigenGWAS for eigenvectors 2–5 for White Leghorn and Dongxiang Blue-shelled chickens.

**Figure 3:**
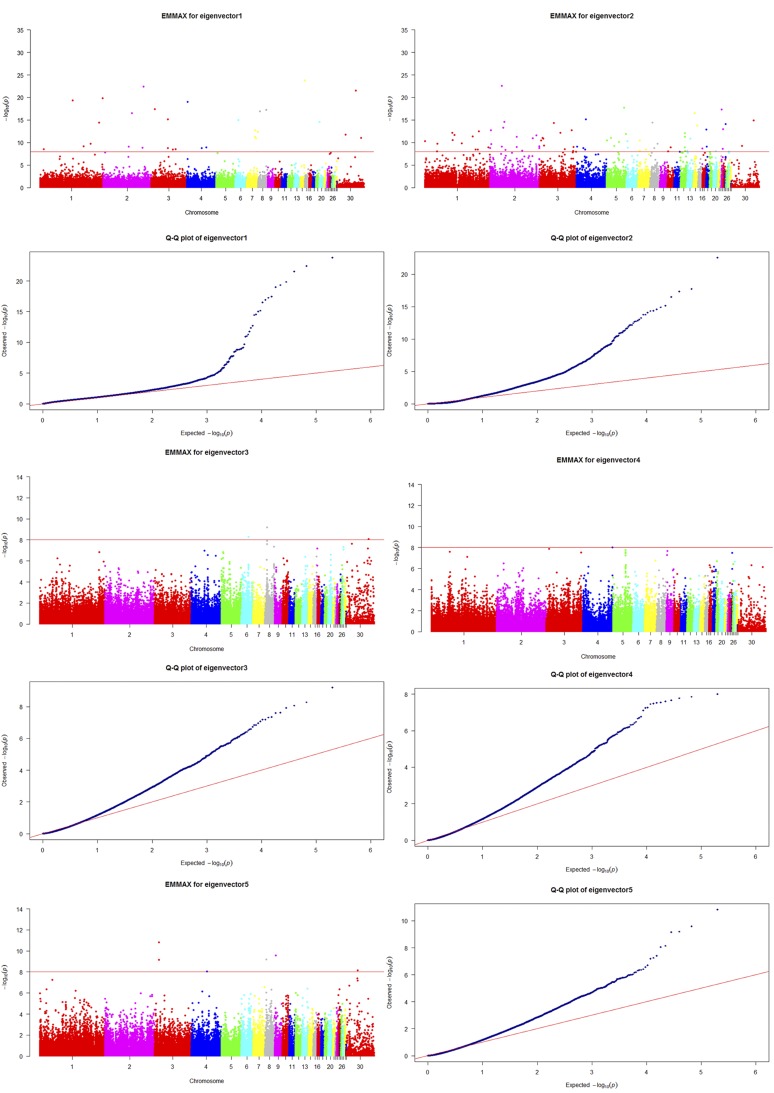
Manhattan and Q-Q plots generated using EMMAX for eigenvectors 1–5 for White Leghorn and Dongxiang Blue-shelled chickens.

We detected a total of 2424 outlier loci in this study, including 2144 positive and 280 negative outliers. These loci correspond to 327 and 94 potentially selected regions, 10.41 and 2.23 Mb in length, and encompassing 0.85 and 0.18% of the chicken genome. It is therefore clear that White Leghorn chickens have undergone more selection, and have been under more pressure, than their counterpart breed. The number, length, and mean of PSRs detected via XP-EHH are shown in Table 1, while the statistics of potentially selected regions on each chromosome are shown in Figure 1C (Manhattan distribution) and Q-Q plots (Figure 1D). We also used EigenGWAS to identify loci under selection among populations for comparison with the XP-EHH method; we used the top five eigenvectors to construct Manhattan plots as shown in Figure 2. We used the top 2–5 eigenvectors for this analysis because the presence of too much noise within the first eigenvector rendered it meaningless for our analysis. We therefore utilized EMMAX to produce Manhattan and Q-Q plots (Figure 3).

**Table 1:** Number and length distribution of PSRs detected using the XP-EHH test (Mb)

CHR	Positive Value			Negative Value			Total SNPs
	SNP1	Sig1	Length (Mean Value)	SNP2	Sig2	Length (Mean Value)	
1	415	90	2,465.755 (27.397)	48	14	314.360 (22.454)	463
2	106	24	623.224 (25.968)	31	14	293.355 (20.954)	137
3	211	31	1,081.652 (34.892)	35	10	246.272 (24.627)	246
4	40	11	241.676 (21.971)	39	14	322.283 (23.020)	79
5	147	21	662.781 (31.561)	5	3	60.020 (20.007)	152
6	111	23	654.200 (28.443)	5	2	40.207 (20.104)	116
7	15	4	99.583 (24.896)	2	2	40.000 (20.000)	17
8	53	9	272.414 (30.269)	5	3	60.293 (20.098)	58
9	316	30	1,171.214 (39.040)	0	0	0	316
10	125	20	654.585 (32.729)	1	1	20.000	126
11	22	7	163.538 (26.363)	11	2	42.212 (21.106)	33
12	19	2	97.988 (48.994)	14	1	83.731 (83.731)	33
13	20	2	75.582 (37.791)	23	6	146.549 (24.425)	43
14	207	18	756.989 (42.054)	12	4	80.214 (20.054)	219
15	192	11	643.978 (58.543)	1	1	20.000 (20.000)	193
16	0	0	0	0	0	0	0
17	14	2	71.710 (35.855)	24	5	172.692 (34.538)	38
18	0	0	0	3	2	40.011 (20.006)	3
19	41	9	248.163 (27.574)	3	2	40.156 (20.078)	44
20	63	7	280.412 (40.059)	4	2	48.901 (24.451)	67
21	7	2	49.305 (24.653)	0	0	0	7
22	0	0	0	0	0	0	0
23	0	0	0	0	0	0	0
24	0	0	0	2	1	20.152 (20.152)	2
25	1	1	20.000 (20.000)	0	0	0	1
26	8	1	20.531 (20.531)	4	2	41.326 (20.663)	12
27	0	0	0	8	3	97.337 (32.446)	8
28	11	2	55.313 (27.657)	0	0	0	11
W	0	0	0	0	0	0	0
Z	0	0	0	0	0	0	0
M	0	0	0	0	0	0	0
32	0	0	0	0	0	0	0
Total	2144	327	10,410.596 (31.84)	280	94	2230.071 (23.72)	2424

SNP1, number of significant SNPs in White Leghorn chickens; SNP2, number of significant SNPs in Dongxiang Blue-shelled chickens; Sig1, number of PSRs in White Leghorn chickens; Sig2, number of PSRs in Dongxiang Blue-shelled chickens.

### Function annotation

Results show that the gene *ROBO1* is significant within potentially selected regions because it acts on follicle development and exerts a marked effect on White Leghorn chicken laying hen performance (Fan 2014). Our results also demonstrate that the gene *OSBP2* functions in cell proliferation (Charman *et al.* 2014), while *SAMSN1* is associated with the malignant hepatocellular carcinoma phenotype (Sueoka *et al.* 2015). We also detected the *ROBO1* and *SAMSN1* genes using the EigenGWAS method, and, in addition to *JARID2*, *RBMS3*, *GPC3*, and *TRIB2*, which are all responsible for immune traits, we isolated genes such as *IGFALS*, which was associated with growth in White Leghorn chickens. Of particular note, EigenGWAS results show that the *SLCO1B3* gene is related to eggshell color (Zheng *et al.* 2014) in Dongxiang Blue-shelled chickens.

In order to further elucidate the biological functions of genes within potentially selected regions, we carried out GO enrichment analyses. Results reveal the presence of six significant KEGG pathways in Dongxiang Blue-shelled chickens, and one significant GO term in the White Leghorn breed (Table 2). Compared to Dongxiang Blue-shelled chickens, selected regions in White Leghorns were mainly enriched in cell metabolism, while, conversely, some amino acids, lipid metabolism, and signal transduction pathways were enriched in the former breed.

**Table 2:** Significant GO or pathway in PSRs

	Category	Term	Count	*p*-Value
White	GOTERM_BP_2	GO:0044237: cellular metabolic process	11	3.2E−2
Green	KEGG_PATHWAY	gga00592: α-linoleic acid metabolism	3	7.6E−3
	KEGG_PATHWAY	gga00591: linoleic acid metabolism	3	8.6E−3
	KEGG_PATHWAY	gga00565: ether acid metabolism	3	2.5E−2
	KEGG_PATHWAY	gga00590: arachidonic acid metabolism	3	2.7E−2
	KEGG_PATHWAY	gga04010: MAPK signaling pathway	6	3.0E−2
	KEGG_PATHWAY	gga00330: arginine and proline metabolism	3	3.8E−2

White, White Leghorn chickens; Green, Dongxiang Blue-shelled chickens.

These differences in amino acid enrichment are indicative of egg quality differences between the two breeds, and to some extent reflect variation between Chinese and western diets.

We established the relationship between PSRs and traits by QTL overlap (Table 3). These data reveal large differences in the number of healthy and production trait selected regions between the two chicken breeds. Indeed, more selected regions for production traits are present in White Leghorn chickens compared to the Dongxiang Blue-shelled breed, likely the result of stronger artificial selection. This result also corroborates the reliability of our method. In addition, the fact that the number of health trait-related selected regions in White Leghorn chickens is also higher than in the Dongxiang Blue-shelled breed is likely the result of greater pressures on the immune system due to the domestic environment since their introduction into China.

**Table 3:** Number of PSRs revealed by QTL analysis in some related traits

Population	Number of PSR (%)	Healthy Characters	Production Traits	Physiological Characters
White	66 (68.75%)	14	49	3
Green	30 (31.25%)	1	29	0

White, White Leghorn chickens; Green, Dongxiang Blue-shelled chickens.

We compared and analyzed information from significant PSRs and the chicken QTL database. Comparisons show that several QTL intervals with lowest *p*-values are due to pathological changes and mineral content in Dongxiang Blue-shelled chickens. However, these intervals are related mainly to tibia length and body weight (*i.e.*, 14, 28, 42, and 70 d), and can help to explain differences in the growth and egg quality traits between the two chicken breeds.

## Discussion

Our PCA results reveal genetic distance between White Leghorn and Dongxiang Blue-shelled chicken breeds. This distance implies trait differences, especially in terms of growth, egg quality, and the immune system.

The phenomenon of LD refers to a nonrandom association between different loci (Single *et al.* 2016), and a long linkage region can result from intensive selection. Thus, understanding LD is necessary if we are to understand the evolutionary history of a population; as shown in Figure 1B, White Leghorn chickens exhibit longer linkage distances compared to the Dongxiang Blue-shelled breed, which may be indicative of relatively higher selection.

We utilized three statistical approaches to detect potentially selected regions in White Leghorn and Dongxiang Blue-shelled chickens in order to achieve explanatory power localizing the source of selection. We initially applied the XP-EHH test to make comparisons between the two chicken breeds. This statistic was originally designed to estimate alleles that have increased in frequency to the point of fixation (or close to it) in one population, as well as to assess haplotype differences between two populations (Simonson *et al.* 2010).

The Dongxiang Blue-shelled breed is particularly renowned for its slow growth compared to White Leghorns. Thus, while the results of our statistical gene comparisons reveal common growth performance in White Leghorn chickens, this is not the case in the Dongxiang Blue-shelled breed, where the insulin-like growth factor (IGF) binding protein acid labile subunit (*IGFALS*) gene was not found in the study.

We also identified a number of genes in this study that are associated with immune system traits in White Leghorn chickens, including *JARID2*, *RBMS3*, *GPC3*, *SARM1*, and *TRIB2*. A previous study has explored the effects and underlying molecular mechanisms of the *JARID2* gene on leukemia cell proliferation (Su *et al.* 2015); the RNA binding motif single stranded interacting protein3 (*RBMS3*) is known to act as a tumor suppressing gene, and is a favorable prognostic marker in lung squamous cell carcinoma. At the same time, the *GPC3* gene inhibits hepatocellular carcinoma cells, and plays an important role in immunity (Dargel *et al.* 2015; Liang *et al.* 2015), while sterile α and TIR motif containing one (*SARM1*) genes are also related to immunity, and are responsible for regulating neuronal inflammation (Lin and Hsueh 2014). Finally, the *TRIB2* gene—a novel regulator of thymocyte cellular proliferation—is important in thymopoietic responses to genotoxic and oncogenic stress, and functions to suppress tumors (Liang *et al.* 2016).

Our results show that there is a much higher number of PSRs in White Leghorn chickens than is the case in the Dongxiang Blue-shelled breed. These results imply that the immune systems of White Leghorn chickens have been subject to significant environmental pressures since being introduced to China because of complex domestic conditions. These chickens also produce more eggs and grow faster as a result of greater selection pressure. Our analysis of PSRs has revealed a series of genes that are associated with important economic traits, and has elucidated molecular genetic mechanisms in both White Leghorn and the Dongxiang Blue-shelled chickens.

## Supplementary Material

Supplemental material is available online at www.g3journal.org/lookup/suppl/doi:10.1534/g3.117.300382/-/DC1.
